# Weight change and the risk of cardiovascular disease in patients with hypertension: A primary-care cohort study

**DOI:** 10.7189/jogh.14.04176

**Published:** 2024-10-01

**Authors:** Zhen Liu, Deliang Lv, Xiaobing Wu, Fengzhu Xie, Qinggang Shang, Wei Xie, Ziyang Zhang, Xiaoxv Yin, Zhiguang Zhao

**Affiliations:** 1 Shenzhen Center for Chronic Disease Control, No. 2021 Buxin Road, Shenzhen, 518020, Guangdong, People’s Republic of China; 2 Department of Social Medicine and Health Management, School of Public Health, Tongji Medical College, Huazhong University of Science and Technology, Wuhan, Hubei, China

## Abstract

**Background:**

Weight control is a cornerstone of hypertension management. Therefore, it is important to understand the relationship of weight change to risk of cardiovascular disease (CVD) among patients with hypertension. We aimed to investigate the association of weight change with the risk of CVD, stroke, and myocardial infarction (MI) among patients with hypertension.

**Methods:**

We obtained the data from medical records of the Hypertension Health Management Program (HMPH) in Shenzhen, China. The present study included 221 454 individuals with hypertension. Weight change over two years was divided into loss ≥10%, loss 5–10%, stable (−5 ~ 5%), gain 5–10%, and gain >10%. Cox regression analyses were applied to assess the associations of weight change groups with the risk of CVD, stroke, and MI.

**Results:**

Compared with the stable weight group (−5 ~ 5%), those with weight loss ≥10% had a higher risk of CVD (hazard ratio (HR) = 1.21; 95% confidence interval (CI) = 1.05–1.40) in the fully adjusted model. Weight gain >10% was significantly associated with a higher risk of CVD (HR = 1.17; 95% CI = 1.04–1.31). In the meanwhile, participants with weight loss ≥10% had significantly higher risks of stroke (HR = 1.20; 95% CI = 1.02–1.41). However, participants with weight gain >10% had an increased risk of MI (HR = 1.45; 95% CI = 1.15–1.82) in the fully adjusted model.

**Conclusions:**

Weight loss or weight gain were associated with higher risks of CVD. Management of patients with hypertension requires close monitoring and appropriate interventions to achieve optimal body weight to prevent adverse outcomes.

Cardiovascular disease (CVD) is the leading cause of death and disability worldwide, with 18.6 million deaths in 2019 [1]. Hypertension is a significant public health burden and an important modifiable risk factor for CVD [2–5]. Therefore, effective health management of patients with hypertension is essential to prevent the development of cardiovascular diseases. Several guidelines for patients with hypertension have been developed in China, which strongly recommend weight management [6].

Current studies have found that weight gain is a risk factor for CVD in patients with hypertension [7,8]. However, the association of weight loss with CVD remains controversial. A study from China showed that hypertensive individuals with weight loss had an increased risk of CVD compared to those with stable weight [7]. A recent study analysed data from 20 737 Chinese patients with hypertension and found that higher body mass index (BMI) variability was associated with a higher risk of CVD among hypertensive patients with weight gain but not in those with weight loss [8]. The different findings may be due to the study design, length of the weight change interval, and the length of follow-up between the weight change and the outcome event [9,10]. In addition, most of the previous studies focused on the general population or people with diabetes, and few studies focused on people with hypertension and were small sample size studies [11–15]. Therefore, further research is urgently required.

A recent study showed that nearly half of Chinese adults aged 35–75 suffered from hypertension, highlighting the heavy burden of hypertension in China [16]. Current recommendations for the prevention and treatment of hypertension and cardiovascular disease emphasise improving disease status and outcomes through weight control [17,18]. Clarifying the association of weight change with CVD in patients with hypertension is helpful in promoting the health management of hypertension. However, there are fewer studies on weight change and CVD in patients with hypertension. In the present study, we aimed to investigate the associations of weight change with the risk of CVD, stroke, and myocardial infarction (MI) among patients with hypertension based on the Health Management Programme of Hypertension (HMPH) of primary-care institutions in China.

## METHODS

This study was approved by the ethics committee of the Shenzhen Center for Chronic Disease Control (Number: SZCCC-2023-032-01-YJ). All participants provided informed consent.

### Study design and data source

The baseline data for this study were derived from medical records of the HMPH in Shenzhen, China. Under the national basic public health service policy, HMPH provides outpatient treatment and follow-up management services for hypertensive patients. According to the HMPH guidelines, residents whose systolic blood pressure (SBP) is ≥140 mm of mercury (mmHg) and/or diastolic blood pressure (DBP) is ≥90 mm Hg for the first time are initially diagnosed with hypertension when factors that may contribute to the increase in blood pressure are excluded, and when all three blood pressure measurements of the day are higher than normal. It is recommended to refer the patient to a higher-level hospital with diagnostic conditions and follow up on the referral results within two weeks. Patients diagnosed with primary hypertension will be enrolled in the HMPH. General practitioners in Shenzhen's 881 community health service centres set up personal health records for hypertensive patients, with regular follow-up visits every three months and free health checks once a year. Baseline data included basic demographic characteristics, physical examination information, lifestyle characteristics, medication information, and biochemical indicators. According to the HMPH, five patients were randomly selected from more than 90% of community health service centers for on-site or telephone quality control to ensure the accuracy of data information. We enrolled 379 898 patients with hypertension from the HMPH between 1 January 2017 and 31 December 2021. The exclusion criteria were as follows:

1) patients with missing or abnormal values for the first and second weight measurements (n = 490);

2) patients with CVD at the time of enrollment (n = 27 352);

3) patients with missing age and sex information (n = 67);

4) patients with missing marital status, education, lifestyle and blood pressure information (n = 4192);

5) patients with missing or abnormal values for fasting plasma glucose (FPG), triglyceride (TG), high-density lipoprotein cholesterol (HDL-C), low-density lipoprotein cholesterol (LDL-C) and blood pressure information (n = 103 208);

6) patients without medication information about antihypertensive drugs, antidiabetic drugs, and lipid-lowering drugs (n = 18 620);

6) patients with abnormal values for weight change (n = 4515). To minimise the interference of outliers, a range of 1–99% of the weight change was used for the analysis. A total of 221 454 patients with hypertension were included in the final analysis (Figure S1 in the **Online Supplementary Document**). This cohort study followed the Strengthening the Reporting of Observational Studies in Epidemiology (STROBE) reporting guideline [19].

### Weight change assessment

We defined the weight change assessment period as two years and all the participants should undergo an assessment period to obtain information on weight change. The first weight value in the health record is considered the baseline weight. Weight change was calculated as the percentage of the difference between baseline weight and two-year follow-up weight ((2-year follow-up weight - baseline weight) / baseline weight ×100%). Based on previous studies, we identified thresholds of 5 and 10% for defining clinical weight change [14,20,21]. Therefore, the participants were categorised into five groups according to the direction of weight change (weight gain vs. weight loss) and percent change in body weight as follows: loss ≥10%, loss 5–10%, stable −5 ~ 5%, gain 5–10%, and gain >10%.

### Study outcomes

Our outcome data were collected through the Shenzhen Cardiovascular and Cerebrovascular Disease Event Monitoring System. The system began to record and report the cardiovascular and cerebrovascular events of Shenzhen residents in 1998 and has a 25-year history of monitoring. The system accuracy rate is kept above 95%, and the missed detection rate is kept below 5%. All CVD incidence data in the monitoring system were derived from hospital admission records at all levels in Shenzhen. The information collected mainly includes basic demographic information, date or basis of diagnosis of stroke or MI, date of death and cause of death, etc. The connection between the monitoring system and the baseline data for this study was matched by a unique ID number. The diagnosis of CVD cases is confirmed through medical imaging examinations such as CT scans or MRI scans by trained clinical staff. The primary outcomes of the study were the first instances of cardiovascular events, specifically incident stroke (I60-64) and MI (I21-22), according to the International Classification of Diseases, 10th revision (ICD-10). In addition, during the annual physical examination of patients with hypertension, patients with hypertension will be asked about their previous medical history/new diseases to ensure the completeness and accuracy of the outcome data. The duration of follow-up was calculated from the date of baseline to the date of the occurrence of CVD, the date of the death diagnosis, or until the end of the follow-up period (15 June 2023), whichever came first.

### Definition of covariates

To gather comprehensive information, data on covariates were obtained from the baseline health checkup. Demographics, lifestyle characteristics, medication information of the patients, and disease information were analysed. Demographics included age, sex (male/female), education level (≤9 years/9–12 years/>12 years), marital status (married/other) and BMI. The calculation method for BMI is the weight in kilograms divided by the height in meters squared, with the unit being kg/m^2^. Lifestyle characteristics included smoking status (current smoker/non-current smoker), drinking status (current drinker/non-current drinker), and physical activity (non-regular exerciser/regular exerciser). Current drinker was defined as someone who drank occasionally, often, or every day. Regular exerciser was defined as someone who exercised at least once a week (≥1 time/week). Medication information included the use of antihypertensive drugs, oral antidiabetic drugs or insulin, and lipid-lowering drugs. Most of the demographics, lifestyle characteristics, and medication information were self-reported. Disease information included the values of SBP and DBP, FPG, TG, HDL-C, and LDL-C. SBP and DBP were calculated as the average of two measurements taken on both arms using a sphygmomanometer after resting for at least five minutes. Morning fasting blood samples from all participants were collected after a minimum of 12 hours of fasting and analysed for biochemical indices including FPG, TG, HDL-C, and LDL-C using a fully automated biochemical analyser.

### Statistical analysis

Baseline characteristics of the participants were described by means ± standard deviation (SD) or median (interquartile range) of continuous variables and frequencies (%) of categorical variables. The subjects were classified according to the weight change categories, and the occurrence of primary endpoint events during follow-up. Additionally, patient characteristics were stratified based on first and second measurements. For the comparison of baseline characteristics between weight change categories, ANOVA and χ^2^ test were used to test the statistical significance of continuous and categorical variables, respectively. The cumulative incidence of endpoint events in the categories of weight change was analysed using the Kaplan-Meier curve, and the differences between the groups were assessed using the log-rank test.

Cox proportional-hazard models were used to calculate the hazard ratios (HRs) and 95% confidence intervals (CIs) between weight change groups with CVD, stroke, and MI. The stable weight (−5 ~ 5%) was considered as the reference. The proportional hazards assumption was assessed using the Schoenfeld residual test, and no significant deviation from the assumption was found. Several models that included different covariates were constructed to estimate HRs and their 95% CI. Model 1 was adjusted for age and sex only; model 2 was further adjusted for BMI, marriage, education, smoking status, drinking status, and physical activity; model 3 was further adjusted for the use of antihypertensive drugs, the use of antidiabetic drugs, the use of lipid-lowering drugs, SBP, DBP, FPG, TG, HDL-C, and LDL-C. The dose-response relationships between the weight change and the probability of outcome occurrence were examined using restricted cubic splines that were adjusted for potential confounding factors. Subgroup analyses and interaction analyses were performed by stratifying the databased on age (<65/≥65 years), sex (male/female), and baseline BMI groups (non-obese: BMI<25 kg/m^2^; obese: BMI≥25 kg/m^2^).

We performed several sensitivity analyses. First, participants who developed CVD within six months of recruitment were excluded to reduce the possibility of reverse causation. Second, participants aged 80 and older were excluded. Third, patients with a BMI<18.5 kg/m^2^ were excluded. Fourth, participants using antidiabetic drugs were excluded. Fifth, we further adjusted for duration of hypertension, waist circumference, and estimated glomerular filtration rate in the models. Sixth, we adopted the missing value processing method of multiple imputation by chained equations based on five replications to impute participants with missing information and repeated all analyses with no loss sample size (351 185 participants).

All statistical analyses were performed using R software version 4.3.1 (R Core Team, Vienna, Austria, http://www.r-project.org). Two-sided *P* < 0.05 was considered statistically significant.

## RESULTS

### Baseline characteristics

The characteristics of the 221 454 individuals with hypertension grouped based on their two-year weight change were listed in **Table 1**. Of all participants, 120 155 (54.26%) were men. The mean (SD) age was 59.69 (11.93) years. The proportion of patients with loss ≥10%, lose 5–10%, stable weight, gain 5–10%, and gain >10% of weight was 3.56, 10.08, 67.76, 10.59, and 8.01%, respectively. In comparison to participants who maintained stable weight, patients with weight gain >10% were more likely to be current smokers and non-regular exercisers. Participants with weight loss ≥10% were older, female, and related to higher baseline BMI levels. Baseline characteristics of participants with and without CVD, stroke, and MI were shown in Tables S1–3 in the **Online Supplementary Document**. Characteristics of patients stratified by first and second measurements are shown in Table S4 in the **Online Supplementary Document**.

**Table 1:** Baseline characteristics of the study population grouped by weight change*

Variables	Total	Loss ≥10%	Loss 5–10%	−5 ~ 5%	Gain 5–10%	Gain >10%	*P*-value
	n = 221 454	n = 7888	n = 22 326	n = 150 050	n = 23 461	n = 17 729	
	100%	3.56%	10.08%	67.76%	10.59%	8.01%	
**Social demographics**							
Age, (mean ± SD)	59.69 (11.93)	61.84 (12.56)	60.81 (11.81)	59.85 (11.79)	58.28 (12.30)	57.85 (12.01)	*P* < 0.0001
Sex							*P* < 0.0001
*Male*	120 155 (54.26)	3715 (47.10)	11 309 (50.65)	82 002 (54.65)	13 018 (55.49)	10 111 (57.03)	
*Female*	101 299 (45.74)	4173 (52.90)	11 017 (49.35)	68 048 (45.35)	10 443 (44.51)	7618 (42.97)	
Marital status							*P* < 0.0001
*Married*	214 838 (97.01)	7554 (95.77)	21 626 (96.86)	145 731 (97.12)	22 724 (96.86)	17 203 (97.03)	
*Other*	6616 (2.99)	334 (4.23)	700 (3.14)	4319 (2.88)	737 (3.14)	526 (2.97)	
BMI, (mean ± SD)	24.96 (3.14)	25.44 (3.25)	25.42(3.15)	25.19 (3.10)	24.33 (3.09)	22.98 (2.66)	*P* < 0.0001
Length of education							*P* < 0.0001
*≤9 y*	126 038 (56.91)	4596 (58.27)	13 158 (58.94)	86 410 (57.59)	12 717 (54.20)	9157 (51.65)	
*9–12 y*	62 721 (28.32)	2201 (27.90)	6059 (27.14)	41 853 (27.89)	7019 (29.92)	5589 (31.52)	
*>12 y*	32 695 (14.76)	1091 (13.83)	3109 (13.93)	21 787 (14.52)	3725 (15.88)	2983 (16.83)	
**Lifestyle characteristics**							
Smoking status							*P* < 0.0001
*Current nonsmoker*	186 935 (84.41)	6867 (87.06)	19 039 (85.28)	126 509 (84.31)	19 713 (84.02)	14 807 (83.52)	
*Current smoker*	34 519 (15.59)	1021 (12.94)	3287 (14.72)	23 541 (15.69)	3748 (15.98)	2922 (16.48)	
Drinking status							*P* < 0.0001
*Current nondrinker*	177 122 (79.98)	6650 (84.31)	18 296 (81.95)	119 535 (79.66)	18 617 (79.35)	14 024 (79.10)	
*Current drinker*	44 332 (20.02)	1238 (15.69)	4030 (18.05)	30 515 (20.34)	4844 (20.65)	3705 (20.90)	
Physical activity							*P* < 0.0001
*Non-regular exerciser*	66 371 (29.97)	2134 (27.05)	6221 (27.86)	44 587 (29.71)	7690 (32.78)	5739 (32.37)	
*Regular exerciser*	155 083 (70.03)	5754 (72.95)	16 105 (72.14)	105 463 (70.29)	15 771 (67.22)	11 990 (67.63)	
**Medication information**							
Any use of antihypertension drugs	214 772 (96.98)	7489 (94.94)	21 390 (95.81)	145 721 (97.11)	22 885 (97.54)	17 287 (97.51)	*P* < 0.0001
Any use of antidiabetic drugs	47 798 (21.58)	2560 (32.45)	6562 (29.39)	31 270 (20.84)	4023 (17.15)	3383 (19.08)	*P* < 0.0001
Any use of lipid-lowering drugs	33 048 (14.92)	1217 (15.43)	3424 (15.34)	22 022 (14.68)	3644 (15.53)	2741 (15.46)	*P* < 0.0001
**Disease characteristics (mean ± SD)**							
SBP (mmHg)	134.96 (14.52)	133.56 (15.17)	133.76 (14.75)	135.05 (14.46)	135.57 (14.48)	135.43 (14.37)	*P* < 0.0001
DBP (mmHg)	82.45 (9.96)	80.22 (9.86)	81.06 (9.86)	82.49 (9.89)	83.34 (10.15)	83.67 (10.16)	*P* < 0.0001
FPG (mmol/L)	6.01 (1.74)	6.16 (2.15)	6.13 (1.93)	6.00 (1.71)	5.93 (1.63)	6.01 (1.71)	*P* < 0.0001
TG (mmol/L)	1.88 (2.38)	1.60 (1.94)	1.71 (2.53)	1.89 (2.38)	2.01 (2.37)	1.99 (2.40)	*P* < 0.0001
HDL-C (mmol/L)	1.32 (1.88)	1.41 (2.12)	1.34 (1.11)	1.32 (1.83)	1.31 (2.04)	1.31 (2.56)	*P* < 0.0001
LDL-C (mmol/L)	2.92 (1.01)	2.82 (1.08)	2.86 (0.99)	2.92 (1.01)	2.95 (1.02)	2.94 (0.99)	*P* < 0.0001

BMI – body mass index, DBP – diastolic blood pressure; FPG – fasting plasma glucose, HDL-C – high-density lipoprotein cholesterol, LDL-C – low-density lipoprotein cholesterol, SBP – systolic blood pressure, TG – triglyceride

*Presented as n (%) unless specified otherwise.

### Risk of cardiovascular disease

During a median follow-up of 2.03(1.78–2.19) years, 4254 participants developed CVD, among which 3377 developed stroke and 920 developed MI. Compared with the stable weight group (−5 ~ 5%), those with weight loss ≥10% had a higher risk of CVD (HR = 1.21; 95% CI = 1.05–1.40) in the fully adjusted model. Weight gain >10% was significantly associated with a higher risk of CVD (HR = 1.17; 95% CI = 1.04–1.31) compared with the stable weight group in the fully adjusted model. In the meanwhile, participants with weight loss ≥10% had significantly higher risks of stroke (HR = 1.20; 95% CI = 1.02–1.41). However, participants with weight gain >10% had an increased risk of MI (HR = 1.45; 95% CI = 1.15–1.82) in the fully adjusted model (**Table 2**).

**Table 2:** Multivariable HR and 95% CI for CVD, stroke, and myocardial infarction of patients with hypertension according to weight change*

Variables	n	No. of events	Model 1	Model 2	Model 3
**Total CVD**					
Loss ≥10%	7888	202	1.25 (1.08–1.44)†	1.23 (1.07–1.42)†	1.21 (1.05–1.40)†
Loss 5–10%	22 326	479	1.11 (1.01–1.22)†	1.10 (1.00–1.21)	1.09 (0.98–1.20)
Stable weight	150 050	2814	1.00 (reference)	1.00 (reference)	1.00 (reference)
Gain 5–10%	23 461	411	0.99 (0.90–1.10)	1.02 (0.92–1.13)	1.01 (0.91–1.12)
Gain >10%	17 729	348	1.15 (1.03–1.29)†	1.21 (1.08–1.36)†	1.17 (1.04–1.31)†
**Stroke**					
Loss ≥10%	7888	163	1.23 (1.05–1.44)†	1.22 (1.04–1.43)†	1.20 (1.02–1.41)†
Loss 5–10%	22 326	369	1.05 (0.94–1.18)	1.05 (0.94–1.17)	1.03 (0.92–1.15)
Stable weight	150 050	2262	1.00 (reference)	1.00 (reference)	1.00 (reference)
Gain 5–10%	23 461	321	0.97 (0.86–1.09)	0.99 (0.88–1.11)	0.98 (0.87–1.10)
Gain >10%	17 729	262	1.08 (0.95–1.23)	1.14 (1.00–1.30)†	1.10 (0.97–1.25)
**Myocardial infarction**					
Loss ≥10%	7888	42	1.32 (0.96–1.80)	1.29 (0.94–1.76)	1.28 (0.93–1.75)
Loss 5–10%	22 326	112	1.29 (1.05–1.58)†	1.27 (1.04–1.55)†	1.26 (1.03–1.54)†
Stable weight	150 050	579	1.00 (reference)	1.00 (reference)	1.00 (reference)
Gain 5–10%	23 461	96	1.13 (0.91–1.40)	1.15 (0.92–1.43)	1.14 (0.92–1.42)
Gain >10%	17 729	91	1.44 (1.16–1.80)†	1.50 (1.20–1.88)‡	1.45 (1.15–1.82)†

BMI – body mass index, CI – confidence interval, CVD – cardiovascular disease, DBP – diastolic blood pressure, FPG – fasting plasma glucose, HDL-C – high-density lipoprotein cholesterol, HR – hazard ratio, LDL-C – low-density lipoprotein cholesterol, SBP – systolic blood pressure, TG – triglyceride

*Model 1: age and sex only; Model 2: BMI, marriage, education, smoking status, drinking status, and physical activity additionally; Model 3: the use of antihypertensive drugs, the use of antidiabetic drugs, the use of lipid-lowering drugs, SBP, DBP, FPG, TG, HDL-C, and LDL-C additionally.

†*P* < 0.05

‡*P* < 0.001

**Figure 1:**
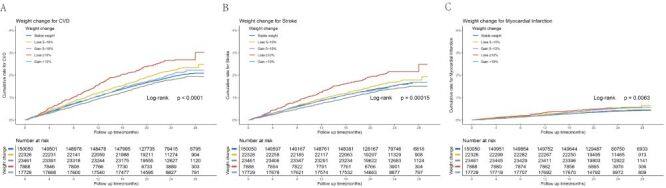
Kaplan-Meier curves are of time to the primary outcome of incident CVD (**Panel A**), stroke (**Panel B**), and myocardial infarction (**Panel C**) by categories of weight change.

**Figure 2:**
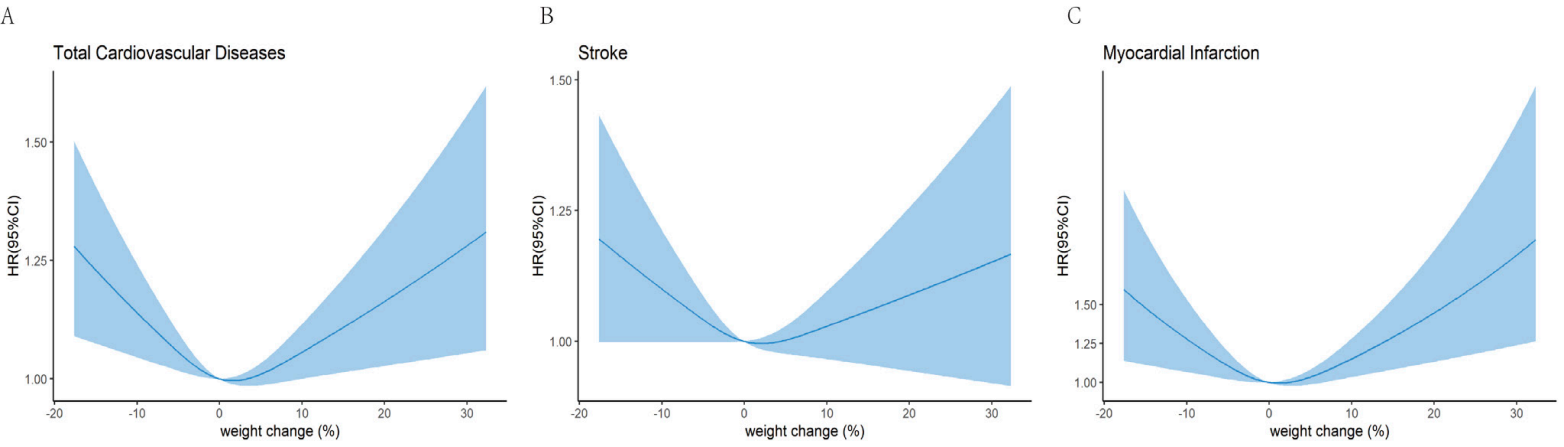
Restricted cubic splines of cox regression showing hazard ratios of incident CVD (**Panel A**), stroke (**Panel B**), and myocardial infarction (**Panel C**) by categories of weight change. Adjusted for age, sex, BMI, marriage, education, smoking status, drinking status, physical activity, the use of antihypertensive drugs, the use of antidiabetic drugs, the use of lipid-lowering drugs, SBP, DBP, FPG, TG, HDL-C, and LDL-C. BMI – body mass index, CI – confidence interval, CVD – cardiovascular disease, DBP – diastolic blood pressure, FPG – fasting plasma glucose, HDL-C – high-density lipoprotein cholesterol, HR – hazard ratio, LDL-C – low-density lipoprotein cholesterol, SBP – systolic blood pressure, TG – triglyceride.

The Kaplan-Meier curve showed that weight change groups were significantly associated with CVD, stroke, and MI (**Figure 1**, panels A–C). When weight change was set as a continuous variable, we observed symmetric V-shaped patterns. Patients with stable weight had the lowest risk of CVD, stroke, and MI, and the risk increased with weight loss or weight gain (**Figure 2**, panels A–C).

### Subgroup analyses

**Figure 3:**
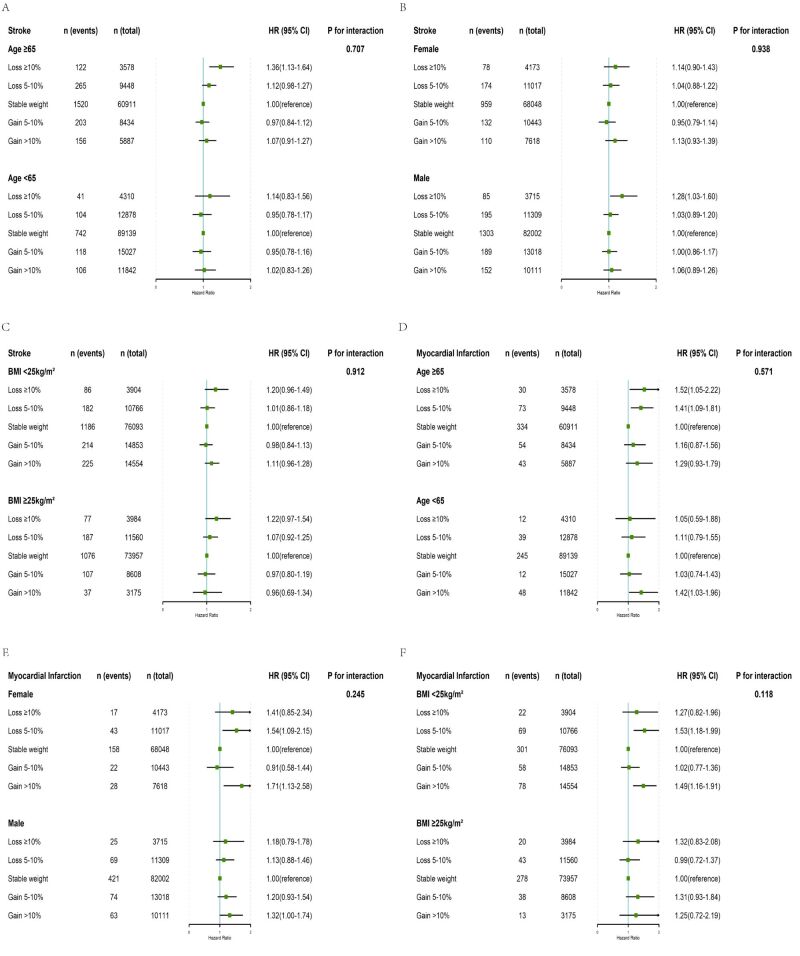
Subgroup analyses on the associations of weight change groups with incident stroke (**Panels A–C**) and myocardial infarction (**Panels D–F**) stratified by age (<65/≥65 years), sex (male/female), and baseline BMI groups (non-obese: BMI<25 kg/m^2^; obese: BMI≥25 kg/m^2^). HR and 95% CI of outcomes by the weight change categories. Adjusted for age, sex, BMI, marriage, education, smoking status, drinking status, physical activity, the use of antihypertensive drugs, the use of antidiabetic drugs, the use of lipid-lowering drugs, SBP, DBP, FPG, TG, HDL-C, and LDL-C. BMI – body mass index, CI – confidence interval, DBP – diastolic blood pressure, FPG – fasting plasma glucose, HDL-C – high-density lipoprotein cholesterol, HR – hazard ratio, LDL-C – low-density lipoprotein cholesterol, SBP – systolic blood pressure, TG – triglyceride.

We conducted stratified analysis and interaction analyses based on age groups (<65/≥65 years), sex (male/female), and BMI groups (non-obese: BMI<25 kg/m^2^; obese: BMI≥25 kg/m^2^) to assess the associations of categories of weight change with the risk of stroke (**Figure 3**, panels A–C) and MI (**Figure 3**, panels D–F) in each subgroup. The risks of stroke and MI showed a U-shaped curve according to weight change in all study subgroups. Females with weight gain >10% had a higher HR for MI (HR = 1.71; 95% CI = 1.13–2.58), and males with weight gain >10% had a higher HR for MI compared with male with stable weight (HR = 1.32; 95% CI = 1.00–1.74). However, there was no interaction between weight change and age, sex, and BMI groups for the analysis of stroke and MI (all *P* for interaction >0.05).

### Sensitivity analyses

When we excluded participants who developed CVD within six months of recruitment, excluded participants aged 80 and older, excluded participants with a BMI<18.5 kg/m^2^, excluded participants using antidiabetic drugs, further adjusted for duration of hypertension, waist circumference, and estimated glomerular filtration rate, or used the multiple imputation, our results remained robust (Figure S2, Table S5 in the **Online Supplementary Document**).

## DISCUSSION

Based on the medical records of the HMPH in China, we examined the associations of weight change with CVD, stroke, and MI among patients with hypertension in a real-world medical setting. Our results showed that hypertensive subjects with weight loss ≥10% or weight gain >10% were significantly associated with increased risks of cardiovascular outcomes compared to those with stable weight after adjustment for BMI and traditional cardiovascular risk factors.

The evidence linking weight gain to a high risk of cardiovascular events in the general population is compelling. A meta-analysis including 14 observational studies and three trials indicated that weight gain was associated with a higher hazard of CVD events compared with stable weight change [12]. In addition, another recent meta-analysis of 23 prospective cohort studies with 1 093 337 participants showed that the five kg increment in body weight could increase the risk of multiple cardiovascular events like stroke and MI [22]. Based on current studies, our study further supported and extended the associations of weight gain >10% with the risk of CVD and MI using a large sample among patients with hypertension. Weight loss unrelated to underlying disease is considered beneficial because being overweight or obese is associated with adverse health outcomes [23]. However, previous studies have shown that weight loss has no benefit for CVD [15,24], and some have even reported that weight loss is associated with a significantly increased risk of CVD and stroke [7,14,25,26]. Based on results from the ADVANCE trial, two-year weight loss >10% was associated significantly with increased risk of major cardiovascular events and CVD mortality compared with stable weight change [27]. Similarly, our study also showed that weight loss ≥10% may increase the risk of CVD and stroke. On the basis of existing studies, this study expanded the existing studies on the association of weight change with CVD in hypertensive patients, providing a theoretical basis and data support for weight management and control in hypertensive patients.

The association of weight gain with CVD may involve certain biological mechanisms. First, weight gain leads to rapid expansion and growth of adipose tissue as a result of changes in metabolism. Metabolically active adipose tissue produces a range of adipokines (e.g. leptin) that lead to adverse outcomes [28]. Second, weight gain promotes the activation of sympathetic pathways [29], leading to increased insulin resistance [30] and further impairment of endothelial, kidney, and heart function, then accelerating the progression of CVD [31]. Additionally, weight gain has been associated with the development of all the components of the metabolic syndrome [32], which was an important risk of CVD. The precise mechanisms that link weight loss and CVD are still not fully clear [13,33,34]. Weight loss may be associated with fat loss as well as muscle loss, which is particularly important in the aging population (sarcopenia). Since muscle mass loss is difficult to recover, weight loss in the elderly is considered undesirable [35,36]. Undiagnosed pre-existing chronic conditions may also be a plausible explanation for the observed increased risk of CVD in the weight loss group, especially for unintentional weight loss [11]. In addition, other possible mechanisms include that weight loss means less nutrient intake, which can lead to anabolic resistance, electrolyte imbalance, insulin resistance, and ultimately CVD [27,37].

To our knowledge, the current study is among the first to investigate the association of weight change with CVD among hypertensive participants. Strengths of this study include the large sample size, prospective study design, and comprehensive sensitivity analysis that supports the findings. However, this study had several limitations. First, due to the relatively short follow-up period, we may observe insufficient cardiovascular outcomes, potentially leading to lower statistical power. Second, our study was observational and cannot establish a causal relationship. Even though we adjusted for confounding variables, we were still unable to reduce the possibility of reverse causation. Third, another important aspect to consider is that the baseline BMI and laboratory indicators were assessed only once, which introduces the possibility of measurement errors. Future studies should consider multiple monitors to reduce this bias. Finally, since this study was conducted exclusively in Shenzhen, the results of this study should be cautious when extending to populations in other countries and regions. Later studies should carry out long-term weight monitoring and follow-up of hypertensive patients across the country, systematically and continuously collect information such as weight, lifestyle, and laboratory measurement indicators, and further explore the association of the trajectory of variables such as weight with CVD.

## CONCLUSIONS

In conclusion, this cohort study of community-dwelling adults with hypertension demonstrates that weight change of loss or gain was related to higher risks of CVD. For the health management of patients with hypertension, the weight and weight change should be monitored regularly and systematically, the weight should be controlled in a relatively stable range, and the sharp increase or decrease in weight should be avoided to prevent the occurrence of CVD.

## Additional material


Online Supplementary Document
