# Supplementary material for: Weight change and the risk of cardiovascular disease in patients with hypertension: A primary-care cohort study
Source: J Glob Health. 2024 Oct 1;14:04176. doi: 10.7189/jogh.14.04176 (PMC11467773; doi:10.7189/jogh.14.04176)
Supplement: Online Supplementary Document [file jogh-14-04176-s001.pdf]

Figure S1 Flowchart for the selection of the study population

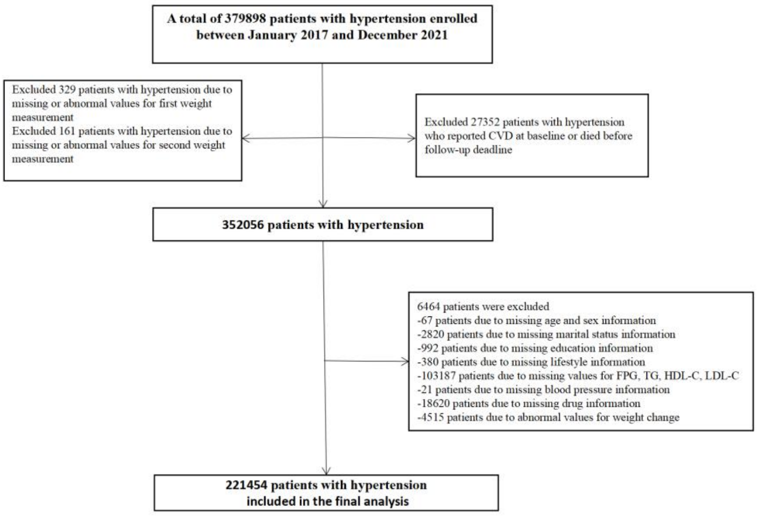

Table S1 Baseline characteristics of patients with and without CVD

	Total (N=221454)	Without CVD (N=217200)	With CVD (N=4254)	*P*-value
**Social demographics**				
Age, (mean±SD)	59.69 (11.93)	59.55 (11.89)	66.91 (11.37)	<0.0001
Gender, n(%)				<0.0001
Male	120155 (54.26%)	117602(54.14%)	2553(60.01%)	
Female	101299 (45.74%)	99598(45.86%)	1701(39.99%)	
Marital status				0.0020
Married	214838 (97.01%)	210745(97.03%)	4093(96.22%)	
Other	6616 (2.99%)	6455(2.97%)	161(3.78%)	
BMI, (mean±SD)	24.96 (3.14)	24.96 (3.15)	24.81 (3.10)	0.0029
Length of education				<0.0001
≤9 years	126038 (56.91%)	123464(56.84%)	2574(60.51%)	
9-12 years	62721 (28.32%)	61608(28.36%)	1113(26.16%)	
>12 years	32695 (14.76%)	32128(14.79%)	567(13.33%)	
**Lifestyle characteristics**				
Smoking status, n(%)				<0.0001
Current nonsmoker	186935 (84.41%)	183522(84.49%)	3413(80.23%)	
Current smoker	34519 (15.59%)	33678(15.51%)	841(19.77%)	
Drinking status, n(%)				0.0142
Current nondrinker	177122 (79.98%)	173783(80.01%)	3339(78.49%)	
Current drinker	44332 (20.02%)	43417(19.99%)	915(21.51%)	
Physical exercise, n(%)				<0.0001
Infrequent exerciser	66371 (29.97%)	65221(30.03%)	1150(27.03%)	
Frequent exerciser	155083 (70.03%)	151979(69.97%)	3104(72.97%)	
**Drug, n(%)**				
Any use of BP lowering treatment	214772 (96.98%)	210688(97.00%)	4084(96.00%)	0.0002
Any use of blood glucose treatment	47798 (21.58%)	46360(21.34%)	1438(33.80%)	<0.0001
Any use of lipid lowering treatment	33048 (14.92%)	32378(14.91%)	670(15.75%)	0.1265
**Disease characteristics**				
SBP(mmHg), (mean±SD)	134.96 (14.52)	134.87 (14.47)	139.26 (16.48)	<0.0001
DBP(mmHg), (mean±SD)	82.45 (9.96)	82.46 (9.94)	81.64 (10.96)	<0.0001
FPG(mmol/L), (mean±SD)	6.01 (1.74)	6.00 (1.72)	6.62 (2.49)	<0.0001
TG(mmol/L), (mean±SD)	1.88 (2.38)	1.88 (2.40)	1.87 (1.59)	0.6918
HDL-C(mmol/L), (mean±SD)	1.32 (1.88)	1.32 (1.87)	1.32 (2.32)	0.9008
LDL-C(mmol/L), (mean±SD)	2.92 (1.01)	2.92 (1.01)	3.01 (1.03)	<0.0001

Data were given as mean±SD, median with interquartile range or n (%).

Abbreviations: CVD, cardiovascular disease; BMI, body mass index; SBP, systolic blood pressure; DBP, diastolic blood pressure; FPG, fasting plasma glucose; TG, triglyceride; HDL-C, high density lipoprotein cholesterol; LDL-C, low density lipoprotein cholesterol.

Table S2 Baseline characteristics of patients with and without stroke

	Total (N=221454)	Without stroke (N=218077)	With stroke (N=3377)	*P*-value
**Social demographics**				
Age, (mean±SD)	59.69 (11.93)	59.57 (11.90)	67.27 (10.99)	<0.0001
Gender, n(%)				0.0014
Male	120155 (54.26%)	118231(54.22%)	1924(56.97%)	
Female	101299 (45.74%)	99846(45.78%)	1453(43.03%)	
Marital status				0.0243
Married	214838 (97.01%)	211584(97.02%)	3254(96.36%)	
Other	6616 (2.99%)	6493(2.98%)	123(3.64%)	
BMI, (mean±SD)	24.96 (3.14)	24.96 (3.14)	24.82 (3.10)	0.0090
Length of education				<0.0001
≤9 years	126038 (56.91%)	123978(56.85%)	2060(61.00%)	
9-12 years	62721 (28.32%)	61832(28.35%)	889(26.33%)	
>12 years	32695 (14.76%)	32267(14.80%)	428(12.67%)	
**Lifestyle characteristics**				
Smoking status, n(%)				0.0007
Current nonsmoker	186935 (84.41%)	184155(84.44%)	2780(82.32%)	
Current smoker	34519 (15.59%)	33922(15.56%)	597(17.68%)	
Drinking status, n(%)				0.0832
Current nondrinker	177122 (79.98%)	174461(80.00%)	2661(78.80%)	
Current drinker	44332 (20.02%)	43616(20.00%)	716(21.20%)	
Physical exercise, n(%)				0.0001
Infrequent exerciser	66371 (29.97%)	65461(30.02%)	910(26.95%)	
Frequent exerciser	155083 (70.03%)	152616(69.98%)	2467(73.05%)	
**Drug, n(%)**				
Any use of BP lowering treatment	214772 (96.98%)	211525(97.00%)	3247(96.15%)	0.0044
Any use of blood glucose treatment	47798 (21.58%)	46651(21.39%)	1147(33.97%)	<0.0001
Any use of lipid lowering treatment	33048 (14.92%)	32495(14.90%)	553(16.38%)	0.0170
**Disease characteristics**				
SBP(mmHg), (mean±SD)	134.96 (14.52)	134.89 (14.47)	139.44 (16.57)	<0.0001
DBP(mmHg), (mean±SD)	82.45 (9.96)	82.46 (9.95)	81.64 (10.92)	<0.0001
FPG(mmol/L), (mean±SD)	6.01 (1.74)	6.00 (1.73)	6.62 (2.50)	<0.0001
TG(mmol/L), (mean±SD)	1.88 (2.38)	1.88 (2.39)	1.85 (1.59)	0.3446
HDL-C(mmol/L), (mean±SD)	1.32 (1.88)	1.32 (1.86)	1.34 (2.58)	0.6473
LDL-C(mmol/L), (mean±SD)	2.92 (1.01)	2.92 (1.01)	2.97 (1.00)	0.0025

Data were given as mean±SD, median with interquartile range or n (%).

Abbreviations: BMI, body mass index; SBP, systolic blood pressure; DBP, diastolic blood pressure; FPG, fasting plasma glucose; TG, triglyceride; HDL-C, high density lipoprotein cholesterol; LDL-C, low density lipoprotein cholesterol.

Table S3 Baseline characteristics of patients with and without MI.

	Total (N=221454)	Without MI (N=220534)	With MI (N=920)	*P*-value
**Social demographics**				
Age, (mean±SD)	59.69 (11.93)	59.66 (11.92)	65.74 (12.53)	<0.0001
Gender, n(%)				
Male	120155 (54.26%)	119503(54.19%)	652(70.87%)	<0.0001
Female	101299 (45.74%)	101031(45.81%)	268(29.13%)	
Marital status				0.0049
Married	214838 (97.01%)	213960(97.02%)	878(95.43%)	
Other	6616 (2.99%)	6574(2.98%)	42(4.57%)	
BMI, (mean±SD)	24.96 (3.14)	24.96 (3.14)	24.80 (3.08)	0.1385
Length of education				0.1024
≤9 years	126038 (56.91%)	125496(56.91%)	542(58.91%)	
9-12 years	62721 (28.32%)	62489(28.34%)	232(25.22%)	
>12 years	32695 (14.76%)	32549(14.76%)	146(15.87%)	
**Lifestyle characteristics**				
Smoking status, n(%)				<0.0001
Current nonsmoker	186935 (84.41%)	186266(84.46%)	669(72.72%)	
Current smoker	34519 (15.59%)	34268(15.54%)	251(27.28%)	
Drinking status, n(%)				0.0268
Current nondrinker	177122 (79.98%)	176413(79.99%)	709(77.07%)	
Current drinker	44332 (20.02%)	44121(20.01%)	211(22.93%)	
Physical exercise, n(%)				0.0455
Infrequent exerciser	66371 (29.97%)	66123(29.98%)	248(26.96%)	
Frequent exerciser	155083 (70.03%)	154411(70.02%)	672(73.04%)	
**Drug, n(%)**				
Any use of BP lowering treatment	214772 (96.98%)	213894(96.99%)	878(95.43%)	0.006
Any use of blood glucose treatment	47798 (21.58%)	47484(21.53%)	314(34.13%)	<0.0001
Any use of lipid lowering treatment	33048 (14.92%)	32923(14.93%)	125(13.59%)	0.2544
**Disease characteristics**				
SBP(mmHg), (mean±SD)	134.96 (14.52)	134.94 (14.51)	138.80 (16.36)	<0.0001
DBP(mmHg), (mean±SD)	82.45 (9.96)	82.45 (9.96)	81.56 (11.17)	0.007
FPG(mmol/L), (mean±SD)	6.01 (1.74)	6.01 (1.74)	6.64 (2.46)	<0.0001
TG(mmol/L), (mean±SD)	1.88 (2.38)	1.88 (2.39)	1.98 (1.63)	0.2267
HDL-C(mmol/L), (mean±SD)	1.32 (1.88)	1.32 (1.88)	1.24 (0.67)	0.1942
LDL-C(mmol/L), (mean±SD)	2.92 (1.01)	2.92 (1.01)	3.17 (1.09)	<0.0001

Data were given as mean±SD, median with interquartile range or n (%).

Abbreviations: MI, myocardial infarction; BMI, body mass index; SBP, systolic blood pressure; DBP, diastolic blood pressure; FPG, fasting plasma glucose; TG, triglyceride; HDL-C, high density lipoprotein cholesterol; LDL-C, low density lipoprotein cholesterol.

Table S4 Characteristics of patients stratified by first and second measurement

	First measurement	Second measurement	*P*-value
Weight(kg),(mean±SD)	65.97(11.05)	66.55(11.48)	<0.0001
BMI(kg/m^2), (mean±SD)	24.96(3.14)	25.40 (3.23)	<0.0001
SBP(mmHg), (mean±SD)	138.07(16.66)	134.96(14.52)	<0.0001
DBP(mmHg), (mean±SD)	84.51(11.74)	82.45(9.96)	<0.0001

Abbreviations: BMI, body mass index; SBP, systolic blood pressure; DBP, diastolic blood pressure.

Figure S2 Sensitivity analysis of association between weight change categories with cardiovascular disease.

Weight change for CVD	No. of events	N	HR (95% CI)	P value
**Sensitive 1**	**3402**	**220242**		
Loss ≥10%			1.20(1.01-1.43)	<0.05
Loss 5-10%			1.11(0.99-1.24)	0.085
Stable weight			1.00(reference)	
Gain 5-10%			1.04(0.92-1.18)	0.491
Gain >10%			1.20(1.05-1.38)	<0.01
**Sensitive 2**	**3779**	**213518**		
Loss ≥10%			1.16(0.99-1.37)	0.074
Loss 5-10%			1.05(0.94-1.17)	0.371
Stable weight			1.00(reference)	
Gain 5-10%			1.03(0.92-1.15)	0.611
Gain >10%			1.19(1.06-1.34)	<0.01
**Sensitive 3**	**4209**	**219474**		
Loss ≥10%			1.21(1.05-1.40)	<0.01
Loss 5-10%			1.09(0.98-1.20)	0.098
Stable weight			1.00(reference)	
Gain 5-10%			1.00(0.90-1.11)	0.946
Gain >10%			1.17(1.04-1.31)	<0.01
**Sensitive 4**	**2816**	**173656**		
Loss ≥10%			1.15(0.94-1.40)	0.164
Loss 5-10%			1.07(0.95-1.22)	0.273
Stable weight			1.00(reference)	
Gain 5-10%			1.07(0.95-1.21)	0.281
Gain >10%			1.19(1.04-1.37)	<0.05
**Sensitive 5**	**4234**	**220462**		
Loss ≥10%			1.17(1.01-1.35)	<0.05
Loss 5-10%			1.07(0.97-1.18)	0.159
Stable weight			1.00(reference)	
Gain 5-10%			0.98(0.88-1.09)	0.743
Gain >10%			1.12(1.00-1.26)	<0.05

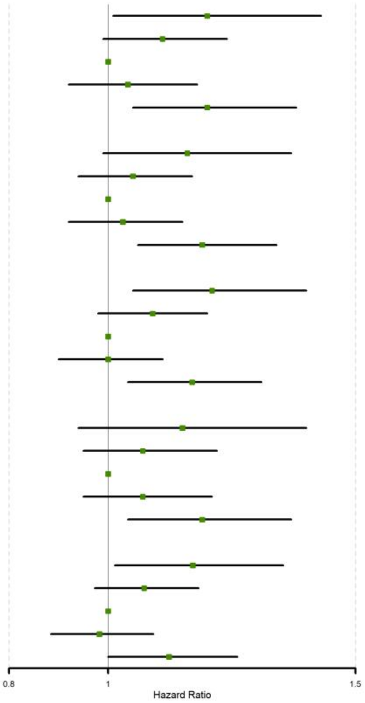

Adjusted for age, sex, BMI, marriage, education, smoking status, drinking status, physical activity, the use of antihypertensive drugs, the use of antidiabetic drugs, the use of lipid-lowering drugs, SBP, DBP, FPG, TG, HDL-C, and LDL-C.

Sensitive 1: Excluding participants with incident CVD <6 months from baseline.

Sensitive 2: Excluding participants aged 80 and older.

Sensitive 3: Excluding participants with a BMI < 18.5 kg/m^2.

Sensitive 4: Excluding participants using antidiabetic drugs.

Sensitive 5: Further adjusting for duration of hypertension, waist circumference and estimated glomerular filtration rate (eGFR) in the models.

Abbreviations: HR, hazard ratio; CI, confidence interval; CVD, cardiovascular disease; BMI, body mass index; SBP, systolic blood pressure; DBP, diastolic blood

pressure; FPG, fasting plasma glucose; TG, triglyceride; HDL-C, high density lipoprotein cholesterol; LDL-C, low density lipoprotein cholesterol.

Table S5 Multivariable HR and 95% CI for CVD, Stroke, and Myocardial infarction of patients with hypertension according to weight change: sensitive analysis 6[a].

	N	No. of events	Model 1	*P*	Model 2	*P*	Model 3	*P*
**Total CVD**								
Loss≥10%	15811	375	1.23(1.10-1.37)	<0.001	1.21(1.08-1.34)	<0.001	1.22(1.10-1.36)	<0.001
Loss 5–10%	33147	664	1.14(1.05-1.23)	<0.05	1.12(1.03-1.22)	<0.05	1.11(1.02-1.21)	<0.05
Stable weight	231424	3917	1.00(reference)		1.00(reference)		1.00(reference)	
Gain 5–10%	37217	622	1.06(0.97-1.15)	0.203	1.08(0.99-1.18)	0.074	1.07(0.98-1.17)	0.113
Gain>10%	33586	596	1.16(1.07-1.27)	<0.001	1.23(1.13-1.35)	<0.001	1.19(1.09-1.30)	<0.001
**Stroke**								
Loss≥10%	15811	286	1.16(1.03-1.31)	<0.05	1.15(1.02-1.30)	<0.05	1.16(1.03-1.31)	<0.05
Loss 5–10%	33147	515	1.11(1.01-1.22)	<0.05	1.10(1.00-1.21)	<0.05	1.09(0.99-1.19)	0.08
Stable weight	231424	3082	1.00(reference)		1.00(reference)		1.00(reference)	
Gain 5–10%	37217	477	1.03(0.94-1.14)	0.513	1.06(0.96-1.16)	0.263	1.05(0.95-1.15)	0.366
Gain>10%	33586	447	1.12(1.01-1.23)	<0.05	1.18(1.07-1.31)	<0.05	1.14(1.03-1.26)	<0.05
**Myocardial infarction**								
Loss≥10%	15811	92	1.43(1.15-1.77)	<0.05	1.38(1.11-1.71)	<0.05	1.37(1.11-1.71)	<0.05
Loss 5–10%	33147	153	1.19(1.00-1.42)	<0.05	1.17(0.99-1.39)	0.072	1.16(0.97-1.37)	0.102
Stable weight	231424	876	1.00(reference)		1.00(reference)		1.00(reference)	
Gain 5–10%	37217	152	1.14(0.96-1.36)	0.128	1.17(0.98-1.39)	0.084	1.17(0.98-1.39)	0.084
Gain>10%	33586	156	1.33(1.12-1.58)	<0.05	1.40(1.18-1.68)	<0.001	1.38(1.16-1.65)	<0.001

Model 1: age and sex only; Model 2: BMI, marriage, education, smoking status, drinking status, and physical activity additionally; Model 3:the use of antihypertensive drugs, the use of antidiabetic drugs, the use of lipid-lowering drugs, SBP, DBP, FPG, TG, HDL-C, and LDL-C additionally.

Abbreviations: HR, hazard ratio; CI, confidence interval; CVD, cardiovascular disease; BMI, body mass index; SBP, systolic blood pressure; DBP, diastolic blood pressure; FPG, fasting plasma glucose; TG, triglyceride; HDL-C, high-density lipoprotein cholesterol; LDL-C, low-density lipoprotein cholesterol.
